# Patterns of weight loss and supplement consumption of male wrestlers in Tehran

**DOI:** 10.1186/1758-2555-3-4

**Published:** 2011-02-12

**Authors:** Ramin Kordi, Vahid Ziaee, Mohsen Rostami, William A Wallace

**Affiliations:** 1 Sports Medicine Research Center, Faculty of Medicine, Tehran University of Medical Sciences, Tehran, Iran; 2 Centre for Sports Medicine, Division of Orthopaedic and Accident Surgery, University of Nottingham, Nottingham, UK

## Abstract

**Background:**

To evaluate the weight loss behavior of male wrestlers in Tehran

**Methods:**

This study was a population-based cross sectional survey. Subjects were 436 wrestlers randomly selected from the wrestling clubs in Tehran employing cluster sample setting method. Subjects were interviewed based on a designed questionnaire. Body fat levels were measured based on skin fold measurements.

**Results:**

Weight loss methods practiced by 62% of all subjects during the previous year employing rapid (≤7 days before the matches) and gradual (>7 days before the matches) weight reduction methods (73% and 34% of wrestlers who reduced their weight respectively). In addition, opinions on weight reduction, the methods of weight loss used, and the side effects of the weight loss practices as well as consumption of supplements among the subjects were reported in this study. The mean percentage of body fat of subjects was 15.9%.

**Conclusions:**

Rapid weight loss for matches and the use of unsafe methods of weight reduction such as fasting, and fluid reduction methods as well as acute side effects of weight loss were prevalent among wrestlers in Tehran. Some preventive measures including education and new rules such as scheduling weigh-ins immediately prior to the competitions and mat-side weigh-in are needed to prevent these unhealthy practices. The weight loss behaviors of these wrestlers should be changed from using dehydration methods to using gradual methods of weight loss.

## Introduction

Weight loss in wrestling has been a concern for researchers and health care professionals for more than half a century [1]. It is reported that wrestlers often practice weight loss in order to be qualified in a lower competitive weight class [1-3]. The new rules and regulations have been partially effective in decreasing unsafe weight loss practices in the USA [4,5].

Fasting and some dehydration methods are the primary methods employed by wrestlers for rapid weight reduction before the matches in the US [6]. Wrestlers have traditionally practiced dehydration as a weight loss method [7]. Dehydration and rapid short-term weight loss can cause various side effects particularly when they are frequently repeated [1]. After the announcement of three deaths because of weight loss for wrestling matches in 1997, several associations in the US instituted new rules which prohibited some methods of weight loss and set a minimum weight limit (based on 5-7% of body fat) [8-10]. None of these rules has yet been enacted in Iran or at the international level. One possible reason for this might be the lack of relevant data from other parts of the world except the US.

Available evidence reported in the literature in this area is mainly based on studies of weight loss behaviors of high school and college wrestlers in the US. Data on weight loss in different international wrestling styles could be found rarely [11]. Evaluation of the pattern of weight loss in Iran could be beneficial to educational and preventive efforts in this country; and to provide new preventive rules at international level.

A greater understanding of weight loss behavior of Iranian wrestlers and their opinion on weight loss is required to develop an effective support system for promoting safe weight loss among these wrestlers. In addition, knowledge of a baseline figure for the percentage of body fat in Iranian wrestlers is essential for planning preventive measures in this area.

The objective of this study was to evaluate the weight loss behavior and the percentage of body fat of general population of wrestlers in Tehran (i.e. wrestlers who were members of the wrestling clubs in Tehran and had at least one year experience of wrestling).

## Materials and methods

This study was a cross sectional survey. Weight loss practice and percentage of body fat of general populations of wrestlers in Tehran was investigated. Of around 20000 wrestlers who met the inclusion criteria of the study, 436 athletes from 28 clubs using the cluster sampling method were recruited. In this regards, Tehran was divided into four zones based on geography and socio-cultural differences. We randomly selected 30% of the clubs in each region and about 15% of the members of each club were included in the study (using the list of registered athletes of the club). Of all randomly selected wrestlers, no one left the study. The researchers based on the designed questionnaire interviewed the subjects.

A questionnaire was designed based on published data in this field and our interview with wrestling experts in Iran to evaluate the weight loss practice of wrestlers. We conducted a test-retest pilot study on 40 wrestlers in two clubs in Tehran with 2 weeks interval. The average reliability coefficient for the questionnaire was good, (Kappa = 0.92, range 0.86 to 1 for different questions and Intra-class correlation coefficient = 0.98, range 0.91 to 1 for different questions). These data suggest that the employed questionnaire is reliable. The questionnaire covered four main following areas: 1) demographic information including current weight and height of wrestlers 2) extent of weight loss, methods and side effects of weight loss; 3) opinion on weight loss and education in this area; 4) consumption of supplements.

To determine the content validity of the questionnaire, a variety of literature on the related topic were reviewed to determine similar areas of emphasis. The questionnaire was also revised by eleven wrestling experts in the area in Iran to ensure its content validity. These experts included wrestlers, coaches, and officials of the "Iranian National Wrestling Institution" and the "National Amateur Wrestling Federation" as well as faculty members of the Faculty of Sports and Physical Education of Tehran University who taught wrestling. The written informed consent was signed by all wrestlers participated in the study. This study was approved by the ethical committee of Tehran University of Medical Sciences.

### Body fat measurement

The percentages of body fat of the subjects were calculated by measuring skin fold thickness. The method recommended by the National Collegiate Athletic Association of the US (NCAA) was employed for the skin fold thickness measurement. Skin-fold thickness was measured in three sites (triceps, subscapular and abdominal [12,13]) and Lohman's equation and Brozek's equation were used to calculate the percentage of body fat. It has been shown that this method is valid and reliable for the estimation of body fat levels in wrestlers [14-17].

Thorland's modification of Lohman's equation that has been validated for college and high school wrestlers was employed to calculate the body density of subjects [13,16,18]. Body density was then converted to percent body fat (%BF) using the Brozek equation that has been validated for wrestlers [12]. A Lafayette skin-fold calliper (Model 01127; Lafayette Instrument Company, Lafayette, IN 47903, USA) was used in the study [15]. The measurements were made by an expert assessor that has enough experience in measuring the skin fold thickness employing caliper.

## Results

**Figure 1:**
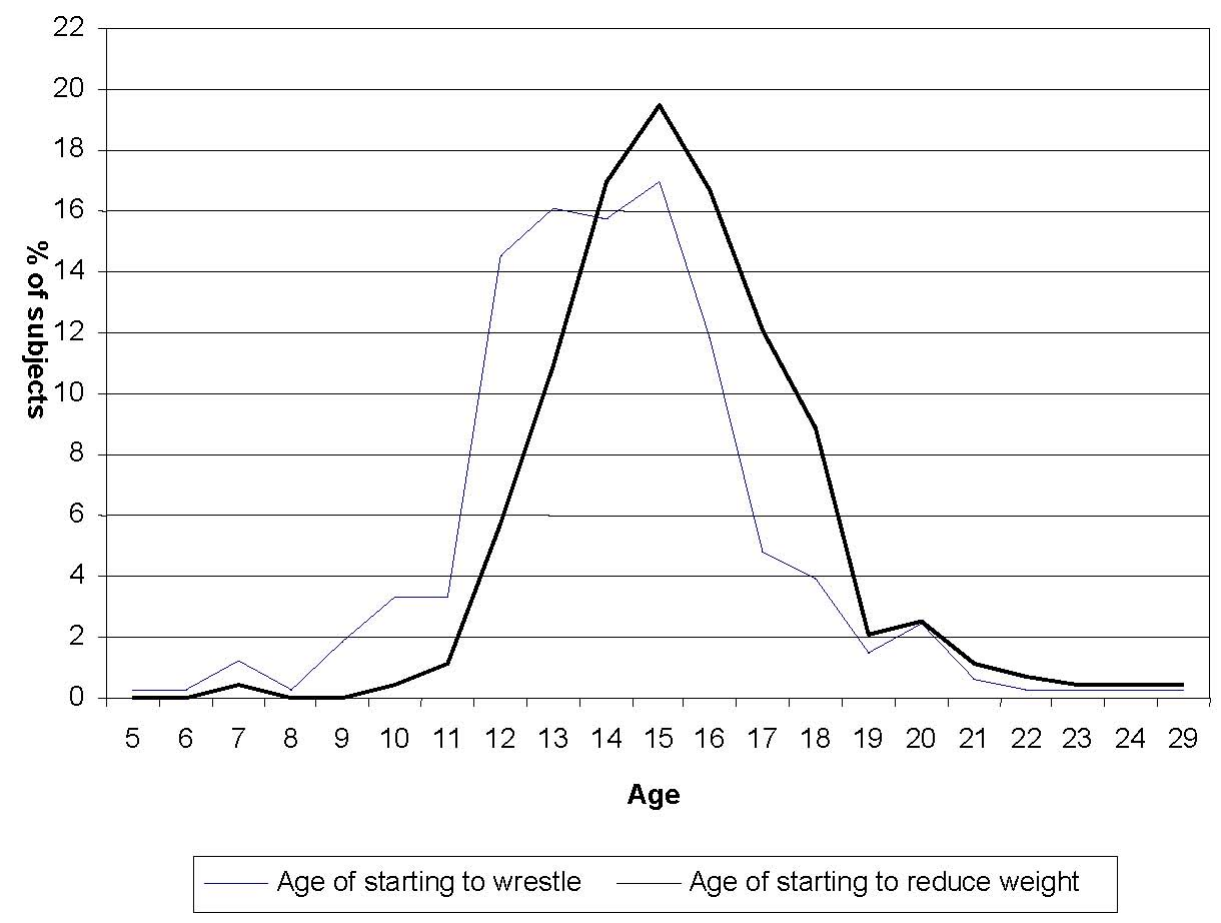
**Age at which subjects started to practice wrestling and started to reduce their weight for wrestling matches**.

According to self-reported demographic data of the wrestlers it was found that subjects (n = 436) had an average age of 18.9 ± 4.1 years (range 11 to 42 years) (Table 1), a mean body weight of 69.6 ± 16.0 kg (Range: 30 to 126 kg) and a mean height of 172 ± 10 cm (Range 130-192 cm). The mean years of experience in wrestling training was found to be 4.0 ± 3.4 years (Range: 1 to 24 years). Subjects started to practice wrestling at a mean age of 14.2 ± 2.8 year (Range: 5 to 29). They reduced their weight for the first time, at a mean age of 15.5 ± 2.4 year (Range: 7-29) (Figure 1). One percent of subjects started to reduce their weight for wrestling before the age of 10 and 18% of the subjects started to reduce their weight before the age of 14. The subjects reduced on average 3.3 ± 1.8 kg (Mode: 3, Range: 0.5-10 kg) of their weight; considering the base weight of wrestlers, it was found that they reduced their weight a mean of 5.0 ± 2.6% of their weight (Range: 1-15%). The weight loss in these wrestlers was performed on average 3.5 ± 2.0 days before the matches (Mode: 2, Range: 1-7).

**Table 1:** The age categories of Wrestlers (n = 436)

Age category (Year)	Number of Wrestlers(Percent)
**<14**	9(2)
**Schoolboys (14-15)**	52(12)
**Cadets (16-17)**	122(28)
**Juniors (18-19)**	153(35)
**Seniors (20 ≤ age <35)**	96(22)
**Veterans (≥ 35)**	4(1)

The majority (75%) of the subjects had reduced their weight for matches at least once before the time of the study. Their last weight loss for matches was on average 9 months before the time of the study (SD: 11, Range 0.5 to 96, Mode: 1). Sixty two percent of the subjects had reduced their weight for matches during the previous year.

Of the subjects who had used weight reduction methods during the previous year, 73% had reduced their weight rapidly before the matches (during 7 days or less), 34% reduced their weight gradually (7% reduced their weight both gradually and rapidly before the matches). In other words 45% of all subjects had used rapid weight reduction techniques before the matches during the previous year. As it is shown in Table 2, they had used this method for weight reduction in variable amounts. Subjects who had episodes of gradual weight reduction for wrestling during the previous year reduced their weight on average by 0.5 ± 0.4 kg/week. This rate was 6.6 ± 6.3 kg/week (about 1 kg/day) for those who used rapid weight loss methods.

**Table 2:** Number of subjects who reduced their body weight by rapid weight loss method during the previous year

Percentage of reduced body weight	Number of subjects (Percent)
**1-2**	28(14)
**3-5**	95(48)
**6-7**	41(21)
**8-9**	24(12)
**≥ 10**	10(5)

### Opinion about weight reduction

Only 28% of the subjects stated that rapid weight loss is not effective in wrestling success. Twenty two percent of the subjects stated that rapid weight reduction is very effective in wrestling success and 50% of the subjects stated that rapid weight reduction is to some extent effective in wrestling success.

Of all the subjects, 22% declared that rapid weight reduction for wrestling has no side effects. Twenty-eight percent of the subjects stated that rapid weight reduction for wrestling can cause short-term side effects (i.e. side effects that affect the wrestler's health during the period of weight reduction) such as fatigue, weakness and depression. Thirty percent of the subjects stated that rapid weight reduction for wrestling can cause long-term side effects (i.e. side effects that affect the wrestler's health in the future) such as impediment normal growth, diminished protein nutritional status and altered hormonal status [1]. Twenty percent of the subjects believed that rapid weight reduction for wrestling can cause both short-term and long-term side effects.

Forty percent of the subjects declared that they had received adequate information about weight loss in wrestling, 33% stated that they had received some information but it was not sufficient, and 27% stated they had received no information about weight reduction in wrestling. The subjects had received information about weight reduction in wrestling from different sources (coach 57%, other wrestlers 28%, parents 6%, physician 3%, Nutritionist 2%, others (such as friends and athletes in other fields) 4%).

For future competitions, 55% of the subjects planned to reduce their weight on average 4.1 ± 2.6 Kg (Range: 1-15) which was accounted for a mean of 5.7 ± 2.9% of their weight (Range: 1.1 to 16.5%).

### Methods of weight loss

The subjects had employed different methods for weight loss (Table 3[2,6,10,19-23]). The subjects who had rapidly reduced their weight for matches during the previous year employed on average 3.4 ± 1.7 methods of weight loss (Mode: 2, Range: 1-9). The majority (72%) of subjects rapidly reduced their weight during the previous year employed at least one fluid reduction method including restricting fluids, using saunas, using a steam room, using rubber/plastic suits and drugs (e.g. diuretics). Exercise and dietary were the most common methods employed by wrestlers for weight loss. Thirty two percent of all subjects employed at least one fluid reduction method during the previous year.

**Table 3:** Weight loss methods employed by our subjects during the previous year and the methods used by wrestlers in the US [2,6,10,19-23]

Category of Methods	Methods of weight reduction	Number of subjects (percent)	Wrestlers in the US
**Exercise**	**Increasing exercise**	301(69)	48 to 97

	**Eating less food in each meal**	257(59)	66 to 73
	
**Dietary**	**Restricting food (skipping 1-2 meals per day)**	257(56)	50 to 77
	
	**Fasting (not eating all day)**	118(27)	8 to 61
	
	**Special dietary regime**	92(21)	_

	**Restricting fluids**	183(42)	17 to 80
	
	**Using saunas**	170(39)	15 to 81
	
**Fluid reduction**	**Using rubber/plastic suits**	153(35)	23 to 49
	
	**Using steam room**	61(14)	_
	
	**Drug, diuretics**	31(7)	0 to 16

**Using Pharmacologic agent except diuretics**	**Other drugs**	9(2)	4 to 10
	
	**Drug, laxative**	9(2)	0 to 18

### Side effects of weight loss

Fifty one percent of wrestlers who had reduced their weight rapidly for matches during the previous year (25% of all subjects) stated that they had experienced on average two side effects of weight loss in this period (Range: 1-12). The average rate of weight loss of these wrestlers was 6.5 kg/week (about 1 kg/day). It was found that fatigue or weakness is the most prevalent side effect among the wrestlers in Tehran (Table 4).

**Table 4:** Percentage of subjects who experienced side effects of weight loss during the previous year

Side effects	Number of subjects (percent)
**Fatigue or weakness**	104 (24)
**Dizziness**	57 (13)
**Muscle cramps**	52 (12)
**Anxiety**	44 (10)
**Headache**	31 (7)
**Myalgia**	26 (6)
**Malaise**	26 (6)
**Nausea**	26 (6)
**Depression**	22 (5)
**Diarrhea**	13 (3)
**Vomiting**	13 (3)
**Palpitation**	13 (3)
**Nose bleeding**	9 (2)
**Feverish**	9 (2)
**Irritability**	9 (2)
**Confusion**	9 (2)
**in-coordination**	9 (2)
**Visited by a physician**	4 (1)
**Syncope**	4 (1)
**Admitted to Hospital**	0 (0)

**Malaise**: an indefinite feeling of debility or lack of health

**Dizziness**: lightheadedness, feeling like you might faint, being unsteady or loss of balance

**Syncope**: temporary loss of consciousness and posture

**Uncoordinated movement**: an abnormality of muscle control or an inability to finely coordinate movements, resulting in a jerky, unsteady, to-and-fro motion of the trunk or the limbs

**Confusion**: the inability to think with your usual speed or clarity

**Irritability**: quick excitability to annoyance, impatience, or anger

**Feverish**: showing symptoms indicating fever (as increased heat and thirst or delirium)

### Consumption of supplements

The majority of subjects (75%) had not consumed supplements or vitamins during the previous six months. The rest of the subjects had consumed supplements (4%), vitamins (12%), or supplements and vitamins (9%). The most commonly used supplements were creatine and protein that had been used by 7% and 3% of all subjects respectively. The most commonly used vitamins were multivitamins, vitamin C and vitamin E that had been used by 8%, 7% and 6% of all subjects respectively (Table 5[20,21,29], Table 6[19-21,30]).

**Table 5:** Supplement consumption by wrestlers in Tehran and the available findings reported from high school wrestlers in US [20,21,29]

Type of Supplement	Percentage of Wrestlers in the US	Percentage of wrestlers in this study
**Vitamins**	28 to 58	21
**Supplements**	59	25

**Table 6:** Percentage of the wrestlers in this study and wrestlers in the US who consumed supplements and vitamins [19-21,30]

Vitamins and Suppliments		Percentage of Wrestlers in this study	Percentage of Wrestlers in US
**Suppliments**			
	Creatine	7	14 - 67
	Protein	3	27 - 30
**Vitamins**			
	Multivitamins	8	40 - 55
	Vitamin C	7	50 - 58
	Vitamin E	6	28 - 33
	Vitamin B Complex	4	28 - 32

### Percentage of body fat

According to the previously mentioned protocol, using the measurements of skin fold thickness of wrestlers, percentage of body fat of the subjects was calculated. The mean percentage of body fat of the wrestlers (Range: 6.3% to 42.7%) was found to be 15.9 (95% confidence Interval: 15.2% to 16.7%). It was also found that only 1.9% of wrestlers have a body fat percentage less than 7.

## Discussion

The results of this study suggest that weight loss for matches is common among wrestlers in Tehran. Sixty-two percent of wrestlers had reduced their weight for matches during the previous year.

### Age

As the pattern of age of wrestlers at which reduced their weight for first time is also shown in figure 1; it was found that the wrestlers started reducing their weight at a mean age of 14.2 ± 2.8 years (range 5 to 29 years) which is higher than the mean age reported for American wrestlers who start training at a mean of 9.4 to 12.3 years in different studies [3,20,24] with an age range from 5 to 16 years [3]. The minimum age to start wrestling in both groups is the same at five years.

Subjects had started to reduce their weight for wrestling at a mean age of 15.5 ± 2.4 (Range: 7 to 29) which is higher than the mean age at which the American wrestlers start to reduce their weight for wrestling which is at age 14 [3,6,21,24].

It is important to note that some of our subjects had started reducing their weight at a very young age. In this regard, 1% of the wrestlers had reduced their weight for the first time before the age of 10.

### Methods of weight loss

Results from this study suggest that employing extreme weight control behaviors including fasting and fluid reduction methods are widely prevalent among wrestlers in Tehran (Table 3).

Except for "gradual dieting", the percentages of other weight reduction methods employed by the subjects in our study are in the range that has been reported by other studies in the US (Table 3). Preventive steps regarding wrestling weight loss in Tehran should focus on changing the weight loss behaviors of wrestlers from using dehydration methods to using gradual methods of weight loss.

A small percentage of our subjects reported that they have employed pharmacological agents to reduce their weight including diuretics (7%), laxatives (2%), and other drugs (2%). Abuse of these drugs is not common. However, due to their potential side effects, particular attention should focus on discouraging wrestlers from using such weight loss methods.

### Body fat in wrestlers

The mean percentage of body fat in high school and collegiate wrestlers in the US as reported by different studies varies from 6% to 12.8% with a reported body fat ranging from 2.4% to 33.7% [1,12,13,16,25,26]. The results of our study show that the percentage of body fat in the members of the wrestling clubs in Tehran (mean 15.9%, 95%CI = 15.2% to 16.7%) are relatively high. This might be because our subjects employed fewer fat reduction methods (i.e. methods that lead to weight reduction of wrestlers through reduction of fat mass of the body) such as exercise and special dietary regime for weight loss.

It is suggested that male wrestlers in the age group of 12 to 24 will not harm themselves if they reduce their body fat down to 7% of their weight [27]. Therefore, it seems that the majority of our subjects could safely reduce their weight by reduction of their body fat. It should be noted that 2% of wrestlers had less than 7% body fat.

### Side effects of weight loss

The results of this study suggest that wrestlers in Tehran commonly experience side effects of rapid weight loss. More than half of the subjects (51%) who had achieved rapid weight reduction for matches during the previous year (about one-fourth (25%) of all subjects) reported that they experienced on average two side effects from the weight loss in this period. Traditionally some of the coaches and wrestlers look at rapid weight loss as a critical component of the "no pain, no gain" philosophy [28]. Sports should aim at increasing the health and well-being of athletes. Sporting governing bodies should try to eliminate suffering, medical risks and pain from those participating in sporting activities.

### Supplements and vitamin

The percentages of wrestlers in this study and wrestlers in the US who consumed supplements and vitamins are shown in the Tables 5 and 6.

As shown in Tables 5 and 6, a comparison of the results of this study with some other studies on wrestlers in the US suggests that consumption of supplements and vitamin among the wrestlers in Tehran is less prevalent than consumption of supplements and vitamins among wrestlers in the US.

### Prevention of weight loss

Weight loss has became part of the culture of wrestling and the athletes are resistant to change [31]. Though it has not been scientifically proven, many coaches and wrestlers in the US believe that wrestlers should practice weight loss to achieve wrestling success [32]. The majority of our subjects (72%) stated that weight loss is important for wrestling success even though the majority of them (77%) believed that rapid weight loss has negative side effects. Therefore, it might be difficult to change the weight loss behavior of wrestlers in Tehran. More than half of the subjects (55%) had plans to reduce their weight by 5.7% for the next competition. Some preventative measures are needed to discourage them from continuing to practice rapid weight loss.

Sixty percent of our subjects stated that they had insufficient information about weight loss. This suggests that wrestlers in Tehran need further education in the area of weight loss.

For high school wrestlers in the US the sources of information about weight loss were mainly coaches (78% to 82%), fellow wrestlers (77%), or former wrestlers (58%). It is reported that 42% to 57% of these wrestlers received help from their parents, and 37% to 41% received assistance from physicians for weight loss [6,20]. Similarly, our subjects had received their information about weight loss in wrestling mainly from coaches (57%) and other wrestlers (28%). This would indicate that the education of coaches is essential in this area.

## Conclusion

In conclusion, it was found that although the rate of weight loss among the wrestlers in Tehran is relatively high they have insufficient information regarding the possible side effects of weight loss. It seems teaching the wrestlers about the healthy methods of weight loss should be performed by their coaches. In addition, employing the fat reduction methods for weight loss could be recommended to wrestlers in Iran, since it was shown that the mean body fat of wrestlers in Iran is higher than US wrestlers. Less consumption of supplements and vitamins by Iranian wrestlers in comparison to the results achieved from other countries might be due to the more traditional beliefs the Iranian wrestlers and their coaches have. The Iranian wrestlers due to their traditional beliefs consume less the synthetic products including vitamins and supplements.

## Competing interests

The authors declare that they have no competing interests.

## Authors' contributions

RK, MR and WAW contributed to the study concept and, with VZ, the study design. RK, MR and VZ were responsible for the acquisition of data. VZ and WAW contributed to the analysis and interpretation of the data. RK and MR drafted the manuscript. RK, WAW and VZ critically revised the manuscript. All of the authors approved the final version of the manuscript submitted for publication.
